# Electrochemical Sensing of Doxorubicin in Breast Cancer Cells Based on Membrane-Permeation Strategy

**DOI:** 10.3390/molecules31060931

**Published:** 2026-03-11

**Authors:** Lizhen Yu, Dandan Wang, Zhongtao Hu, Xuefeng Hou, Shuxue Wang, Wenzhi Zhang, Lihua Li

**Affiliations:** 1 Drug Research and Development Center, School of Pharmacy, Wannan Medical College, Wuhu 241000, China; yulizhen@wnmc.edu.cn (L.Y.); 20239148@stu.wnmc.edu.cn (D.W.); 20239160@stu.wnmc.edu.cn (Z.H.); houxuefeng@wnmc.edu.cn (X.H.); 23107070019@stu.wnmc.edu.cn (S.W.); 2 Anhui Provincial Engineering Laboratory for Screening and Re-Evaluation of Active Compounds of Herbal Medicines in Southern Anhui, Wannan Medical College, Wuhu 241002, China; 3 Anhui Provincial Engineering Research Center for Polysaccharide Drugs, Wannan Medical College, Wuhu 241002, China; 4 Anhui Innovative Center for Drug Basic Research of Metabolic Diseases, Wannan Medical College, Wuhu 241002, China

**Keywords:** doxorubicin, electrochemical sensing, MCF-7 cells, iRGD, membrane permeation

## Abstract

Monitoring the concentration of doxorubicin (DOX) was critical for tumor treatment, but existing methods failed to cross cell membrane. Here, an electrochemical platform for intracellular DOX detection in MCF-7 cells based on membrane-permeation strategy was developed. A modified gold electrode was prepared via electrodepositing AuNPs and assembling SH-DNA. Concurrently, the silica nanosphere/gold nanocluster-circular transmembrane peptide (SiO_2_/AuNCs-iRGD) composite nanoparticles with membrane permeability, tumor targeting, and imaging capability were synthesized. After co-incubation of SiO_2_/AuNCs-iRGD with MCF-7 cells and DOX, followed by co-incubation with the DNA-modified electrode, intracellular DOX intercalated into the DNA backbone, and redox-generated electrons were transferred to the electrode to produce a concentration-correlated electrochemical signal. The modification of the electrode, the morphology of the composite nanoparticles and the detection process were characterized by means of SEM, TEM, CV, EIS, DPV, fluorescence spectroscopy and laser confocal imaging. Under the optimized conditions, the proposed method exhibited a wide detection range of 0.05–300 μmol/L, with a detection limit of 0.01 μmol/L. Moreover, the modified electrode demonstrated satisfactory regenerability, and the proposed method showed excellent reproducibility and stability. The development platform could offer a new strategy for real-time assessment of drug concentration within cultured breast cancer cells in vitro.

## 1. Introduction

Chemotherapy remains a cornerstone of clinical oncology, with therapeutic efficacy critically governed by intracellular accumulation of chemotherapeutic agents [1,2,3]. Doxorubicin (DOX), a widely administered anthracycline antibiotic, demonstrates broad-spectrum antitumor activity against breast cancer, leukemia, and other malignancies [4,5,6]. As a chemotherapy drug, a single-dose intravenous infusion concentration of DOX is 60–75 mg/m^2^. It exerts antitumor effects mainly by interfering with the synthesis of DNA and RNA within cells [7]. The cytotoxic effect of DOX on tumor cells depends on the drug concentration within the cells. Therefore, the determination of the DOX concentration within tumor cells can provide important information for the treatment of tumors, the progression of the disease, and the quality of life of patients. However, current clinical administration lacks real-time pharmacodynamic monitoring. There is a critical need for analytical platforms that can quantify the intracellular drug concentration to optimize the therapeutic outcomes.

Established analytical techniques, including high performance liquid chromatography (HPLC) [8], fluorescence spectroscopy [9,10], and flow cytometry [11], enable the quantitative determination of DOX. However, these approaches necessitate cell lysis, suffer from poor temporal resolution, and provide limited spatial information. These limitations underscore the demand for label-free, real-time analytical approaches that preserve cellular integrity while providing molecular-level insights.

Electrochemical biosensing represents a powerful paradigm for cellular analysis, which can convert physiological activities within cells into electrical signals for detection [12,13]. Due to rapidity, ultra-high sensitivity, excellent temporal resolution and minimal sample preparation requirements, this technique is widely applied for the detection of both small and large biomolecules [14,15,16]. Significant progress has been witnessed in extracellular monitoring in this field [17,18], but intracellular analysis remains challenging because of the presence of the cell membrane barrier [19]. Current methods are difficult to identify and accurately obtain the electrochemical signals of substances within living cells across the cell membrane. Innovative strategies that bridge intracellular compartments with electrode interfaces are urgently needed to achieve accurate and quantitative detection of drug concentrations within cells.

Integrin-targeting peptides offer a promising solution to this interfacial challenge. The RGD transmembrane peptide is a short peptide composed of three amino acids: arginine, glycine and aspartic acid [20]. It is widely present in the body and can specifically bind to the overexpressed integrin αvβ3 and αvβ5 in tumor cells and mediate various pathological and physiological processes [21,22,23]. Among them, the iRGD peptide has a ring structure, including the RGD sequence and the C-terminal sequence (Cend R) [24,25]. The iRGD peptide exhibits dual functionality: its RGD motif binds αvβ3 and αvβ5 integrins overexpressed on tumor cells, while its CendR sequence mediates neuropilin-1-dependent internalization [26,27,28]. The unique combination enables iRGD to achieve specific targeting against tumors and enhance cell uptake, making it highly suitable for the development of advanced biological analysis interfaces [29]. Based on these characteristics, when iRGD is modified onto the fluorescent composite nanoparticles, it not only enhances the tendency and targeting ability of the nanoparticles towards tumor cells but also improves their permeability across the membrane. Moreover, it enables cell imaging, providing new ideas and methods for the diagnosis and treatment of tumors.

In this study, iRGD-modified composite fluorescent nanoparticles (SiO_2_/AuNCs-iRGD) were prepared as the “key” to open the cell membrane. After breast cancer cells were treated with SiO_2_/AuNCs-iRGD, the permeability of the cell membrane increased, allowing the DNA molecules on the surface of the modified electrode to enter the cells and bind with DOX inside the cells [30]. Subsequently, the electrons generated by the oxidation-reduction reaction of DOX were transferred along the DNA backbone to the electrode surface, generating quantifiable electrical signals proportional to intracellular drug concentration. This enabled the quantitative detection of DOX concentration within the cells. Simultaneously, the fluorescent properties of SiO_2_/AuNCs enabled optical imaging, thereby providing the spatial validation of cellular localization and viability [26]. The detection mode addressed fundamental challenges in intracellular analysis by: (1) establishing a direct electrochemical connection across cellular membranes, (2) enabling real-time, non-destructive drug monitoring, and (3) providing multimodal verification through complementary optical imaging.

## 2. Results and Discussions

### 2.1. Characterization of the AuNPs/Au Electrode

To enhance the sensitivity of analyte detection, electrode modification was indispensable. Compared with the method of directly coating AuNPs on the electrode, electrode deposition to generate AuNPs had the advantages of strong bonding, high stability, good conductivity, controllable morphology, large specific surface area, and fast electron transfer rate [31]. It was significantly superior to the traditional coating method and was more suitable for constructing highly sensitive electrochemical sensors [32]. After incubating the AuNPs/Au electrode with the SH-DNA solution for 16 h, a specific Au-S was formed between them [33]. This specific covalent binding not only firmly anchored the SH-DNA molecules on the AuNPs layer but also ensured the orientation of DNA molecules, laying a foundation for their subsequent biological functions.

**Figure 1:**
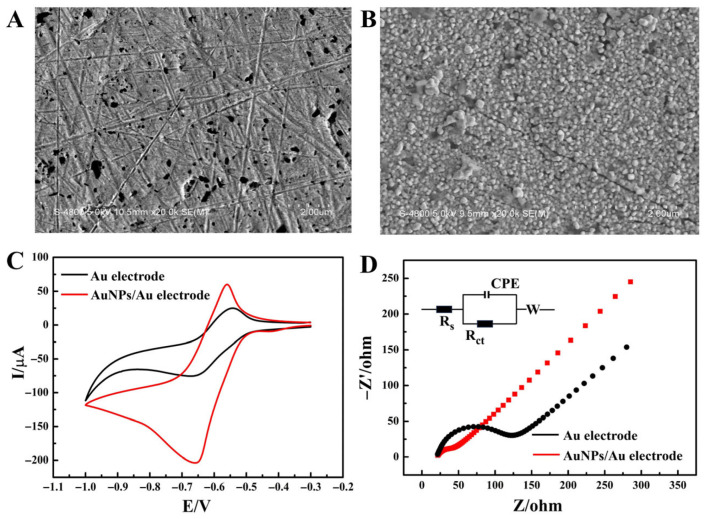
SEM images of (**A**) the Au electrode and (**B**) the AuNPs/Au electrode. (**C**) CV curves of the Au electrode and the AuNPs/Au electrode. (**D**) EIS curves of the Au electrode and the AuNPs/Au electrode (inset: equivalent electrical circuit).

In this study, in order to investigate the effect of gold electrodes before and after the electrodeposition of AuNPs on the sensitivity of DOX detection, an SH-DNA-modified Au electrode or an SH-DNA-modified AuNPs/Au electrode was incubated in 50 μM of DOX solution for 2 h, and CV was determined in Tris-HCl buffer solution (pH 7.4). Meanwhile, the EIS measurement was carried out in a 5 mM [Fe (CN)_6_]^3−/4−^ solution. Figure 1C shows that the reduction peak potential of DOX cyclic voltammetry occurred at −0.58 V. After the electrodeposition of AuNPs, the peak current signal significantly increased, and the impedance decreased (Figure 1C,D). This phenomenon was attributed to AuNPs improving the specific surface area of the electrode (Figure 1B), thereby enabling the modification of more SH-DNA and the binding of more DOX molecules. Additionally, AuNPs could facilitate electron transfer and enhance the conductivity of the electrode [34]. Therefore, the AuNPs/Au electrode showed a higher peak current and a very small impedance value (the equivalent circuit-fitting parameters obtained from EIS are shown in Table S1).

### 2.2. Characterization of SiO_2_/AuNCs-iRGD

**Figure 2:**
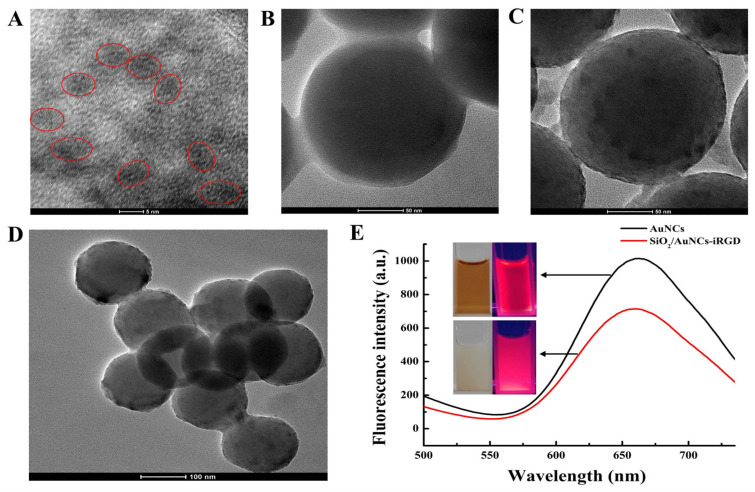
TEM images of (**A**) AuNCs, (**B**) NH_2_-SiO_2_NPs, (**C**) SiO_2_/AuNCs, and (**D**) SiO_2_/AuNCs-iRGD. (**E**) Fluorescence spectrum of SiO_2_/AuNCs-iRGD (inset: photographs of AuNCs and SiO_2_/AuNCs-iRGD suspension under natural light and UV light).

The BSA-protected gold nanoclusters synthesized in the laboratory had a diameter of approximately 5 nm (Figure 2A). BSA molecules restricted Au_25_ nanoclusters within their internal hydrophobic cavities through the coordination and reduction effects of amino acid residues, forming a “core–shell” structure (Au_25_ as the core and BSA as the shell). This distribution mode could effectively prevent the aggregation of AuNCs through the steric hindrance effect of BSA [26,35]. When excited at 380 nm, AuNCs emitted red fluorescence, with the maximum emission wavelength at 660 nm. The surface of AuNCs was rich in carboxyl groups (derived from BSA). Under the activation of EDC/NHS, these carboxyl groups underwent an amide reaction with the amino groups on the surface of the NH_2_-SiO_2_ nanoparticles, thereby fixing AuNCs on the surface of the silica nanoparticles and generating SiO_2_/AuNCs. At the same time, during the formation process of SiO_2_/AuNCs, BSA provided dual protection for AuNCs through Au-S bonds (covalent interaction) and hydrophobic encapsulation (non-covalent interaction), enabling them to maintain fluorescence stability over a wide pH range, high salt concentrations, and various buffer systems such as HEPES and PBS [35] and preventing their aggregation during the conjugation with SiO_2_NPs. In contrast to the TEM image of NH_2_-SiO_2_NPs (Figure 2B), the TEM image of SiO_2_/AuNCs exhibited numerous, individual, dark “islands”, indicating that AuNCs was distributed homogeneously on the surface of SiO_2_NPs (Figure 2C), which was consistent with reports in the literature [36]. To further confirm the effective binding of AuNCs with SiO_2_NPs, an SEM-EDS mapping test of SiO_2_/AuNCs was conducted. The results showed that, in addition to Si elements, Au and S elements also appeared in the sample (Figure S1), and these two elements could only come from BSA protected-AuNCs. In the presence of NHS and EDC, SiO_2_/AuNCs were conjugated to iRGD via amide bonds, resulting in the formation of SiO_2_/AuNCs-iRGD (Figure 2D). As the SiO_2_ nanoparticles powder was white, the synthesized SiO_2_/AuNCs-iRGD suspension appeared pale white under sunlight and red under ultraviolet light. Compared with AuNCs, the fluorescence intensity of SiO_2_/AuNCs-iRGD decreased, and the maximum emission wavelength shifted to 655 nm (Figure 2E).

To investigate whether iRGD and SiO_2_/AuNCs was effectively connected, a comparative experiment was conducted (showed in electronic Supplementary Material). The results were displayed in Figure S2. Due to the fact that iRGD had active targeting (capable of specifically binding to the highly expressed integrin on the surface of MCF-7 cells) and transmembrane effects, this property was endowed to SiO_2_/AuNCs-iRGD, resulting in a greater accumulation of SiO_2_/AuNCs-iRGD in MCF-7 cells and displaying strong red fluorescence (Figure S2B). However, SiO_2_/AuNCs did not possess this effect (the BSA on the surface of AuNCs could not specifically bind to the integrin), merely showing the passive penetration effect of nanoparticles into tumor cells. Therefore, only a small amount of SiO_2_/AuNCs could enter MCF-7 cells (Figure S2A), which confirmed the binding of iRGD to SiO_2_/AuNCs.

### 2.3. The Assembly Process of Cells on the Electrode Surface

**Figure 3:**
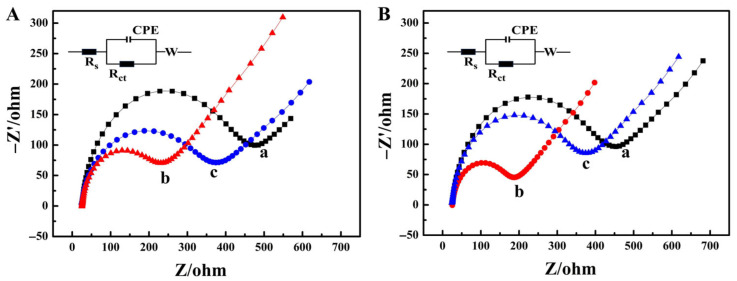
EIS of DNA modified AuNPs/Au electrode (a) before and (b) after incubation with (**A**) MCF-7 cells and 50 μM DOX, and (**B**) MCF-7 cells, SiO_2_/AuNCs-iRGD (containing 100 μM iRGD), and 50 μM DOX. Curve (c) corresponds to further treatment with cell lysis buffer. The inset figures show the equivalent circuits in Figure 3A,B.

EIS was used to characterize the assembly process of the electrode surface. Since DNA had an electron-shielding effect and hindered the transfer of electrons [37]. On the other hand, DNA carried a negative charge and repelled [Fe (CN)_6_]^3−/4−^; thus, the DNA-modified AuNPs/Au electrode exhibited a relatively high impedance (Figure 3A-a). When it interacted with intracellular DOX, the impedance decreased (Figure 3A-b). This was attributed to the fact that the positive charge of DOX neutralized the negative charge of DNA, thereby reducing the repulsion between [Fe (CN)_6_]^3−/4−^ and DNA [38]. When the modified electrode was further acted upon by the cell lysis solution, the cells on the electrode surface were lysed, and the DNA molecules were exposed again, resulting in an increase in impedance (Figure 3A-c).

After the modified electrode was incubated with the cell suspension containing SiO_2_/AuNCs-iRGD, due to the good affinity of SiO_2_/AuNCs-iRGD to the cells and its membrane-penetrating property, the cell permeability increased, facilitating the easier entry of DNA molecules on the electrode surface into the cell interior. In addition, the cells were fixed on the electrode surface through the DNA chain, shielding the negative charge of DNA, resulting in a reduction in impedance. However, the impedance reduction cannot be fully restored by the action of the cell lysis solution (Figure 3B).

### 2.4. Evaluation Results of Intracellular Detection Method for DOX

**Figure 4:**
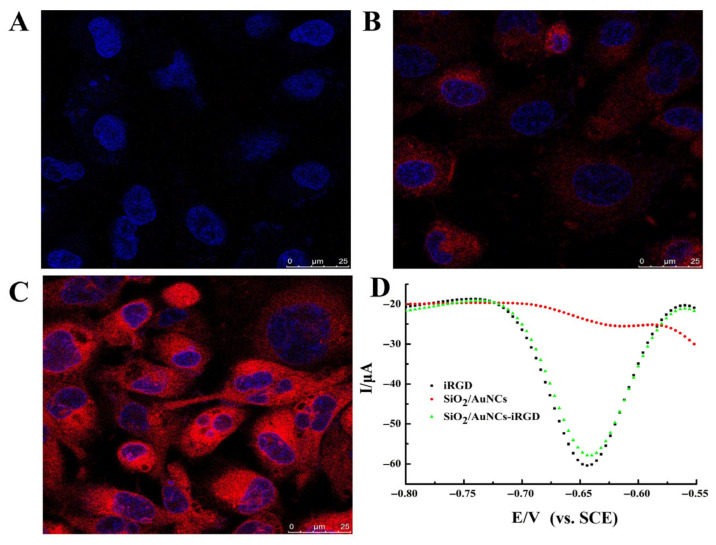
(**A**) Laser confocal image of MCF-7 cells treated with iRGD and 50 μM DOX. (**B**) Laser confocal image of MCF-7 cells treated with SiO_2_/AuNCs and 50 μM DOX. (**C**) Laser confocal image of MCF-7 cells treated with SiO_2_/AuNCs-iRGD and 50 μM DOX. (**D**) DPV curves obtained after incubating the above samples with the modified electrode.

In order to determine whether the detected electrochemical signals originated from intracellular DOX, several comparative experiments were conducted. The results shown in Figure 4 indicated that almost no red fluorescence was observed in the samples with iRGD, but the current signal peak was relatively large. This was because iRGD itself could not emit red fluorescence, but it had membrane permeability and active targeting properties, which could drive DOX into the cells. The samples containing SiO_2_/AuNCs showed a small amount of red fluorescence, but the peak current was very weak. This was attributed to the passive targeting property and certain affinity of SiO_2_/AuNCs to cells [39,40]. However, without iRGD modification, its ability to penetrate the cell membrane was poor. Therefore, a small amount of red light could be observed around the cells, but DOX hardly entered the cells from the cell suspension. After the sample was rinsed with PBS, the drug was almost completely removed; SiO_2_/AuNCs-iRGD combined the advantages of both iRGD and SiO_2_/AuNCs. The sample appeared to have strong red fluorescence and a relatively large peak current, which not only helped DOX in the extracellular environment enter the cell, but also owned the function of fluorescence imaging.

Based on the above experiments, we further investigated whether the exogenously added DOX could be fully internalized by the cells. The results showed that the detectable DPV signal increased with the elevation of DOX concentration. However, when the final concentration reached 400 μM, the DPV signal entered a plateau stage, and electrochemical signals could be detected in the washed solution. This indicated that exogenously added DOX could be completely internalized by the cells at a final concentration below 400 μM.

### 2.5. Conditions Optimization of Experimental Conditions

**Figure 5:**
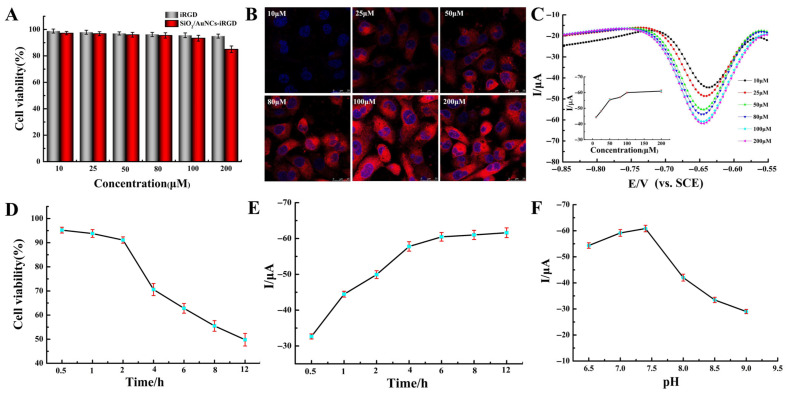
(**A**) The survival rate and (**B**) laser confocal images of MCF-7 cells after incubation with different concentrations of SiO_2_/AuNCs-iRGD for 2 h. (**C**) DPV of the DNA-modified AuNPs/Au electrode with MCF-7 cells, 50 μM DOX, and different concentrations of SiO_2_/AuNCs-iRGD together (inset: calibration curve for DPV values). (**D**) The effect on the survival rate of MCF-7 cells after incubation with SiO_2_/AuNCs-iRGD (containing 100 μM iRGD) for different times. (**E**) The influence of incubation time of the DNA-modified electrode with MCF-7 cells, SiO_2_/AuNCs-iRGD (containing 100 μM iRGD) and 50 μM DOX on the DPV signal intensity. (**F**) The influence of buffer solution pH on DPV signal intensity.

To achieve better detection sensitivity, several detection conditions were optimized. Firstly, the concentration of SiO_2_/AuNCs-iRGD added to the cell suspension was investigated. As can be observed from Figure 5A, the concentration of SiO_2_/AuNCs-iRGD ranged from 10 to 100 μM and had a negligible effect on cell survival. When the concentration of SiO_2_/AuNCs-iRGD reached 200 μM, the cell survival rate dropped below 85%. Meanwhile, the laser confocal imaging results showed that as the concentration of SiO_2_/AuNCs-iRGD increased, more SiO_2_/AuNCs-iRGD was taken up by the cells, and the observed red fluorescence became more prominent, as shown in Figure 5B. On the other hand, a gradual increase in the concentration of SiO_2_/AuNCs-iRGD also led to an increase in cell permeability, which in turn promoted the specific interaction between DOX and DNA. As a result, the DPV signal also became stronger, as shown in Figure 5C. Based on the above results, 100 μM of SiO_2_/AuNCs-iRGD was selected for the subsequent experiments.

The incubation time of SiO_2_/AuNCs-iRGD in the cell suspension had a significant impact on cell viability. As shown in Figure 5D, the longer the incubation time, the poorer the cell activity. After more than 2 h, the cell survival rate was very unsatisfactory. Similarly, we also optimized the incubation time after inserting the DNA-modified electrode on the intensity of the DPV signal. The results showed that the signal intensity basically reached a stable state after 6 h of incubation (Figure 5E). Therefore, 2 h and 6 h were chosen as the incubation time for the co-culture of SiO_2_/AuNCs-iRGD with cells and the incubation time for the co-culture of the modified electrode with cells, respectively.

Moreover, the influence of the pH value of the Tris-HCl buffer solution on the intensity of the electrochemical detection signal was investigated. The results in Figure 5F revealed that the DPV signal intensity increased gradually within the pH range of 6.5 to 7.4, whereas it decreased progressively when the pH exceeded the physiological range. This could be attributed to the alkaline conditions reducing cell activity [41], thereby affecting the signal transduction between DNA and doxorubicin.

### 2.6. Electrochemical Quantitative Detection of Intracellular DOX

**Figure 6:**
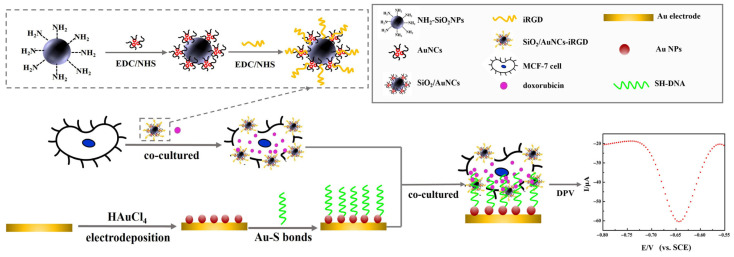
A schematic illustration of the electrochemical detection strategy for DOX concentration in breast cancer cells.

**Figure 7:**
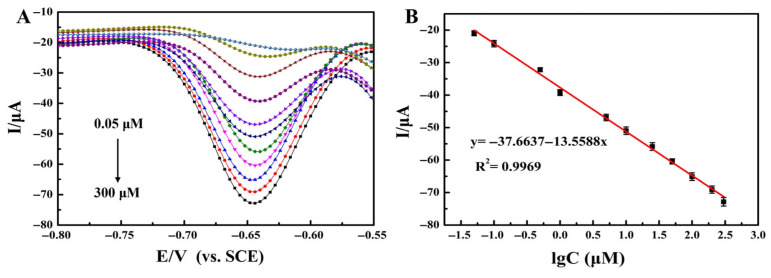
(**A**) DPV responses for the quantitative detection of intracellular DOX concentration. (**B**) The calibration plot of the proposed method for DOX detection. Error bars represent the standard deviations from five replicate measurements.

Under optimized experimental conditions, quantitative detection of intracellular DOX was carried out according to the strategy in Figure 6. As can be seen in Figure 7A, the DPV signal intensity increased gradually with the increasing DOX concentrations in the range of 0.05–300 μmol/L, and a good linear relationship was presented between the two variables. The fitted linear equation was y = −37.6637 − 13.5588lg C, R^2^ = 0.9969 (Figure 7B), with a detection limit of 0.01 μmol/L.

The developed method for quantitatively detecting the intracellular concentration of DOX in MCF-7 cells had three core advantages compared to previous studies (Table 1). First, an extensive detection range: the detection range (0.05–300 μmol/L) covered all clinical treatment scenarios and solved the problem of “concentration compatibility”. Second, an ultra-low detection limit: the detection limit of 0.01 μmol/L had reached the level of trace-level detection. Third, the non-destructive detection of cells in vitro: in this study, the detection of drugs in MCF-7 cells was achieved under mild cell compatibility conditions (pH 7.4, 37 °C), without the need to lyse the cells or use toxic reagents. It allowed for the real-time monitoring of dynamic changes in the DOX concentration within cultured breast cancer cells (e.g., accumulation and metabolic processes over 0–24 h post-administration).

### 2.7. Detection Process and Possible Detection Mechanisms

The steps and mechanisms of the electrochemical platform for quantitatively detecting intracellular DOX in MCF-7 cells based on the membrane permeation strategy are summarized in Figure 6. (1) The Au electrode was subjected to surface modification. AuNPs were loaded onto the surface of electrode through electrodeposition technology. The SH-DNA was then firmly fixed on the electrode surface by Au-S chemical bonds, and an electrochemical sensing substrate was constructed. Here, AuNPs not only provided more binding sites for DNA, but also enhanced electron transfer efficiency [47]. This, in turn, provided fundamental structural support for the electron transfer along the DNA backbone from the DOX redox reaction to the electrode surface, enabling the transmission of electrical signals. (2) The targeted nanocomposites were prepared. Fluorescent nanocomposites SiO_2_/AuNCs were prepared and the tumor-targeting penetrating peptide iRGD was coupled to its surface to obtain the SiO_2_/AuNCs-iRGD nanocomposites. By leveraging the specific affinity and targeted recognition ability of the iRGD peptide towards tumor cells, SiO_2_/AuNCs-iRGD could precisely locate and enrich breast cancer cells, enabling the detection system to specifically act on target cells and avoid interference from non-tumor cells, significantly enhancing the targeting and specificity of the detection. Meanwhile, the membrane penetration function of the iRGD peptide worked in synergy with the material properties of SiO_2_/AuNCs, effectively increasing the permeability of the breast cancer cell membrane [48,49]. This provided a crucial guarantee for the transmembrane entry of SH-DNA fixed on the electrode surface into the interior of the cells, breaking the membrane barrier limitation for the detection of intracellular substances. Furthermore, SiO_2_/AuNCs-iRGD retained the inherent fluorescence properties of SiO_2_/AuNCs, enabling simultaneous optical imaging monitoring of cell localization, distribution, and activity status. It complemented electrochemical quantitative detection and achieved a comprehensive and reliable detection result through the collaborative process of “target enrichment- transmembrane transport-signal transduction-imaging verification”. (3) Cell pre-treatment and co-culture were performed. MCF-7 cells were incubated with SiO_2_/AuNCs-iRGD nanocomposite materials and different concentrations of DOX simultaneously, allowing the nanocomposite materials to fully interact with the cells and enabling DOX to enter the cells. (4) Electrochemical signal transduction and quantitative analysis were performed. The DNA-modified electrodes were immersed in the pre-treated cell suspension for incubation. With the enhanced cell membrane permeability, the single-stranded DNA fixed on the electrode surface was transported into the interior of MCF-7 cells. The intracellular DOX was specifically bound to the DNA chain through an embedding effect, forming a DOX-DNA complex [38,50]. The electrons generated by the redox reaction of DOX were efficiently transferred along the DNA molecular backbone to the electrode surface, achieving the transfer of electrons across the cell membrane; this process converted the chemical information of intracellular DOX into quantifiable electrical signals, and the intensity of the electrical signals was positively correlated with the concentration of intracellular DOX, thereby enabling the quantitative detection of intracellular DOX in MCF-7 cells.

### 2.8. The Regeneration of the Modified Electrode and the Reproducibility and Stability of the Detection Method

**Figure 8:**
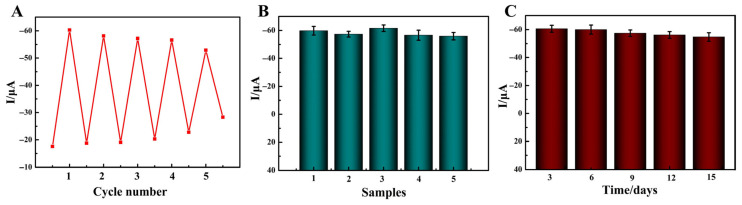
(**A**) The regenerative performance of the modified electrode. (**B**) The reproducibility and (**C**) stability of the detection method.

To quantify the regenerative capability of the modified electrode, the same electrode was used for repeated detection of intracellular DOX at the same concentration (50 μM). After each detection cycle, the electrode was regenerated according to the previously described procedure, and DPV signal intensity was recorded. The experimental results (Figure 8A) showed that the DPV signal intensity of the biosensor remained at 87.7% of the initial value after five regeneration cycles, with an RSD of 4.7%, which fully confirmed that the designed regeneration process could effectively restore the electrochemical activity of the electrode and the modified electrode had good regeneration ability. However, when the regeneration cycle number reached six, the DPV signal intensity dropped to less than 80% of the initial value, which could not meet the detection accuracy requirements. New modified electrodes needed to be prepared. Figure 8B presents the experimental results of the reproducibility of our development method, showing that the DPV signal intensities of the five modified electrodes were highly consistent. The calculated RSD value was 4.1%. Furthermore, the experimental results of the stability study indicated that the DPV value decreased by 9.7% after 15 days compared to the initial reaction (Figure 8C). The above results suggested that the developed detection method possessed satisfactory reproducibility and stability.

## 3. Materials and Methods

### 3.1. Reagents and Instruments

Doxorubicin was purchased from Solebao Technology Co., Ltd. (Beijing, China). iRGD (90% purity) was customized by China Peptides Co., Ltd. (Shanghai, China). Potassium ferricyanide, gold chloride (HAuCl_4_), N-hydroxysuccinic acid amide (NHS), and 1-ethyl-(3-dimethyl-aminopropyl)-carbodiimide hydrochloride (EDC) were purchased from Aladdin Chemistry Co., Ltd. (Shanghai, China). Mercapto DNA was purchased from Beijing Baiao Leibo Technology Co., Ltd. (Beijing, China). Dulbecco’s Modified Eagle Medium (DMEM) and Ethylenediaminetetraacetic acid (EDTA) were purchased from Shanghai McLean Bio-Chemical Technology Co., Ltd. (Shanghai, China). MCF-7 cells were purchased from Shanghai Fuheng Bio-Tech Co., Ltd. (Shanghai, China) and cultured at 37 °C with 5% CO_2_.

The morphology of the electrode and nanoparticles were characterized by a scanning electron microscope (SEM; Hitachi S-4800, Tokyo, Japan) and transmission electron microscopy (TEM; JEM-1200EX, JEOL, Tokyo, Japan). Cell imaging was observed by laser confocal microscopy (TCS-SP8, Leica, Wetzlar, Germany) in 405 nm and 633 nm laser excitation samples and photographed under a 63 x oil lens.

All electrochemical measurements, including electrochemical cyclic voltammetry (CV), electrochemical impedance spectroscopy (EIS) and differential pulse voltammetry (DPV), were conducted on a CHI660E electrochemical workstation (Chenhua Instrument Co., Ltd., Shanghai, China) equipped with a three-electrode system comprising a modified gold electrode (working electrode), a saturated calomel electrode (reference electrode) and a platinum-wire electrode (auxiliary electrode).

### 3.2. Modification of Gold Electrode

Before modification, the gold electrode was pretreated according to the method described in the literature [51]. The treated clean gold electrode was immersed in 1.0 mg/mL of HAuCl_4_ solution containing 0.1 M KNO_3_ and electrodeposited at −0.2 V for 20 s to obtain the electrode modified with gold nanoparticles (AuNPs/Au) [34,52]. The AuNPs/Au electrode was then incubated in 1 μM of SH-DNA solution for 16 h, so that the DNA was fixed on the surface of the electrode by the Au-S bond between SH-DNA and AuNPs.

### 3.3. Preparation of SiO_2_/AuNCs-iRGD

The preparation of BSA-protected AuNCs, NH_2_-SiO_2_ NPs, and SiO_2_/AuNCs was carried out according to our previous method [26,48,49]. The obtained SiO_2_/AuNCs were ultrasonically dispersed in 2 mL of PBS buffer solution (pH 7.0), followed by the addition of appropriate amounts of NHS and EDC. The mixture was stirred for 30 min for activation. Subsequently, 4 mg of iRGD was added into the above mixture, stirred for 24 h, and then washed three times with PBS to acquire the target SiO_2_/AuNCs-iRGD nanocomposites.

### 3.4. Assembly of Cells on the Electrode Surface

Human breast cancer MCF-7 cells were cultured in complete DMEM in a humidified incubator with 5% CO_2_ at 37 °C [53]. After cell expansion, they were digested with trypsin containing EDTA for 5 min, centrifuged at 1000 rpm for 5 min, and the cell concentration was adjusted with DMEM to 1 × 10^6^ cells/mL for use. Subsequently, 100 μL of SiO_2_/AuNCs-iRGD (containing a final iRGD concentration of 100 μM) and 100 μL of DOX solution (with a final concentration of 50 μM) were added to the above cell suspension. The mixture was incubated at 37 °C for 2 h, then centrifuged to remove the supernatant; the resulting precipitate was rinsed with PBS and transferred to the corresponding test tubes. Next, 900 μL of culture medium was added, and the supernatant was removed after swirling for 5 min. Subsequently, 300 μL of medium was added to each tube to resuspend the cells, resulting in a cell suspension sample. For a parallel experiment, 300 μL of trypsin was added to each well for cell digestion and lysis. Finally, the DNA-modified AuNPs/Au electrode was inserted into the cell suspension and incubated at 37 °C for 6 h to dynamically monitor the impedance changes.

### 3.5. Evaluation of Intracellular Detection Methods for DOX Concentration

The concentration of the MCF-7 cell suspension was adjusted to 1 × 10^6^/mL and divided into nine portions. Among them, three portions were added with the iRGD solution (with a final peptide concentration of 100 μM), another three portions were added with the SiO₂/AuNCs suspension (with a nanoparticle concentration of 100 μg/mL), and the remaining three portions were added with the SiO₂/AuNCs-iRGD suspension (containing 100 μM iRGD). DOX was then added to all nine cell suspensions with a final concentration of 50 μM. The samples were incubated at 37 °C for 2 h and subsequently centrifuged to collect the precipitate, which was washed three times with PBS. Next, 900 μL of culture medium was added to the precipitate, and the mixture was centrifuged for 5 min, after which the supernatant was discarded. Each tube was then resuspended with 300 μL medium. Some cell suspension samples were taken for laser confocal imaging. The DNA-modified AuNPs/Au electrodes were inserted into the remaining cell suspension and incubated at 37 °C for 6 h to perform the electrochemical detection.

### 3.6. Optimization of Detection Conditions

To investigate the effect of the developed method on electrochemical detection of intracellular DOX in MCF-7 cells, several key experimental parameters were optimized, including the dosage of SiO_2_/AuNCs-iRGD, the co-incubation time of SiO_2_/AuNCs-iRGD with cells, the incubation time of the modified electrode with the cell suspension, and the pH value of the buffer solution used for electrochemical detection [54]. The optimization results are presented in Section 2.5.

### 3.7. Electrochemical Detection of DOX in Cells

According to the optimized experimental conditions described in Section 2.5, 100 μL of SiO_2_/AuNCs-iRGD (containing 100 μM iRGD) was co-incubated with MCF-7 cells and DOX at a series of concentrations (0.05, 0.1, 0.5, 1.0, 5.0, 10.0, 25.0, 50.0, 100.0, 200.0, 300.0 μM), followed by DPV measurement to evaluate the feasibility of electrochemical detection for intracellular DOX.

### 3.8. Evaluation of the Regenerative Properties of the Modified Electrode and the Reproducibility and Stability of the Detection Method

The regeneration of electrodes was crucial for extending their service life, reducing the cost of testing, and enabling an efficient, green and long-term use of electrochemical detection. Based on the detection mechanism of this experiment, the regeneration steps were as follows: after the modified electrode completed one cycle of intracellular DOX detection, it was immediately taken out of the cell suspension and gently rinsed with pre-cooled PBS buffer (pH 7.4) three times to remove adsorbed cell debris, residual culture medium, unbound DOX and other substances on the electrode surface. Subsequently, the electrode was immersed in Tris-HCl buffer (10 mM, pH 8.5) containing 0.1 M NaCl and incubated at 37 °C for 30 min. The high ionic strength and weak alkaline environment can weaken the hydrophobic interactions and hydrogen bonds between DOX and DNA, thereby promoting the dissociation of DOX from the DNA strands. The electrode was removed from the regeneration solution, and its surface was gently rinsed with ultrapure water three times and soaked for 5 min to remove the residual regeneration solution, desorbed DOX, and a small amount of loosely adsorbed impurities. After regeneration treatment, the electrode was immersed in a 1.0 mM [Fe (CN)_6_]^3−/4−^ solution, and CV scanning was performed. Regeneration was regarded as successful if the peak shape was symmetric and the peak current deviated by ≤10% from that of the freshly prepared modified electrode; the electrode can then be reused for the next detection. Otherwise, it should be discarded.

The repeatability of the detection method was investigated by simultaneous parallel experiments with five temporary prepared electrodes to detect 50 μM DOX in MCF-7 cells. DPV signal intensities of five electrodes were recorded, and the relative standard deviation (RSD) was calculated.

To assess the stability of the detection method, several DNA-modified AuNPs/Au electrodes were prepared and stored in a 4 °C refrigerator. Under the same experimental conditions, DOX in breast cancer cells was detected every 3 days, and the DPV values were recorded.

## 4. Conclusions

In this study, the relationship between the drug concentration in cultured breast cancer cells (an in vitro cell model) and the electrochemical signal responses was explored. Using DOX as a model drug and SiO_2_/AuNCs-iRGD as a targeted “molecular key”, the targeting and membrane-penetrating properties of this nanocomposite facilitated the specific interaction between the SH-DNA immobilized on the electrode and intracellular DOX levels in cancer cell models. This interaction was transduced extracellularly and converted into quantifiable electrochemical signals, thus enabling the accurate quantitative detection of intracellular DOX under the in vitro conditions used in the work.

Notably, the developed sensing platform demonstrated excellent performance with core quantitative characterization indicators as follows: a wide detection range of 0.05–300 μmol/L for intracellular DOX in MCF-7 cells, a low detection limit of 0.01 μmol/L, and a good linear relationship between the DPV signal intensity and DOX concentration with the fitted linear equation y = −37.6637 − 13.5588lg C (R^2^ = 0.9969). Additionally, the modified electrode possessed satisfactory regenerability, retaining 87.7% of the initial DPV signal intensity after five regeneration cycles with an RSD of 4.7%. The detection method also showed excellent reproducibility (RSD = 4.1% for five modified electrodes) and stability (only a 9.7% decrease in DPV signal intensity after 15 days). Overall, this developed detection strategy expanded the application scope of electrochemical analytical methods in biomedical research and provided a novel paradigm for the quantification of intracellular drugs in cancer cell models.

## Figures and Tables

**Table 1:** A comparison of methods for the detection of DOX present in the literature.

Method	Linear Range (μmol/L)	LOD (μmol/L)	Applications	Reference
High performance liquid chromatography	0.086–0.86	0.043	Saliva	[42]
Colorimetry	33–48	0.013	Human urine and serum	[43]
Fluorometry	0–20	0.01	Human serum	[44]
Electrochemistry	100–600	63.5	Human serum	[45]
Localized Surface Plasmon Resonance	0–10	0.42	not reported	[46]
Electrochemistry	0.05–300	0.01	Intracellular	This work

## Data Availability

The original contributions presented in this study are included in thearticle. Further inquiries can be directed to the corresponding authors.

## Supplementary Materials

The following supporting information can be downloaded at https://www.mdpi.com/article/10.3390/molecules31060931/s1, Figure S1: SEM-EDS mapping images of SiO_2_/AuNCs; Figure S2: Laser confocal images of MCF-7 cells after incubation with SiO_2_/AuNCs (A) and SiO_2_/AuNCs-iRGD (B) for 2 h at 37 °C; Table S1: Equivalent circuit fitting parameters obtained from EIS for different samples.
