# Supplementary material for: Electrochemical Sensing of Doxorubicin in Breast Cancer Cells Based on Membrane-Permeation Strategy
Source: Molecules. 2026 Mar 11;31(6):931. doi: 10.3390/molecules31060931 (PMC13029333; doi:10.3390/molecules31060931)
Supplement: Supplementary file 1 [file molecules-31-00931-s001.zip › molecules-4150439-supplementary.pdf]

molecules

Supplementary Material

Electrochemical Sensing of Doxorubicin in Breast Cancer Cells Based on Membrane-Permeation Strategy

Lizhen Yu [1,2,†], Dandan Wang [1,†], Zhongtao Hu [1], Xuefeng Hou [1,2,3,4], Shuxue Wang [1], Wenzhi Zhang [1,2,3,4,*] and Lihua Li [1,2,3,4,*]

1 Drug Research and Development Center, School of Pharmacy, Wannan Medical College, Wuhu 241000, China; yulizhen@wnmc.edu.cn (L.Y.); 20239148@stu.wnmc.edu.cn (D.W.); 20239160@stu.wnmc.edu.cn (Z.H.); houxuefeng@wnmc.edu.cn (X.H.); 23107070019@stu.wnmc.edu.cn (S.W.)
2 Anhui Provincial Engineering Laboratory for Screening and Re-Evaluation of Active Compounds of Herbal Medicines in Southern Anhui, Wannan Medical College, Wuhu 241002, China
3 Anhui Provincial Engineering Research Center for Polysaccharide Drugs, Wannan Medical College, Wuhu 241002, China
4 Anhui Innovative Center for Drug Basic Research of Metabolic Diseases, Wannan Medical College, Wuhu 241002, China
* Correspondence: zhangwz@wnmc.edu.cn (W.Z.); llh@wnmc.edu.cn (L.L.)
† These authors contributed equally to this work.

1. SEM-EDS mapping images of SiO_2/AuNCs

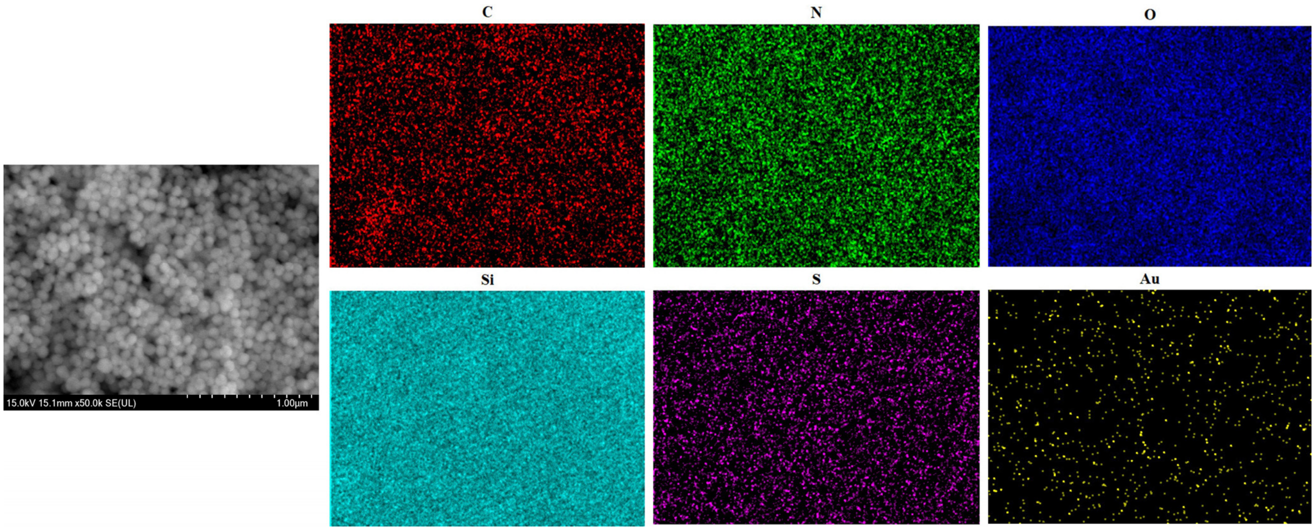

Figure S1. SEM-EDS mapping images of SiO_2/AuNCs.

2. Confirmation of conjugation of iRGD to SiO_2/AuNCs nanoparticles

To investigate whether iRGD and SiO_2/AuNCs was effectively connected, a comparative experiment was conducted. The concentration of the MCF-7 cell suspension was adjusted to 1×10^6/mL and divided into six portions. Three portions were added with 100μL of SiO_2/AuNCs suspension (the concentration of nanoparticles was 100μg/ml), and the other three portions were added with the same volume and concentration of the SiO_2/AuNCs-iRGD suspension. The samples were incubated at 37 ℃ for 2 h, then centrifuged to collect the precipitate, which was washed with PBS three times. Next, 900 μL of culture medium was added to the precipitate, centrifuged for 5 min, and the supernatant was discarded. Each sample was then resuspended with 300 μL of culture medium and subjected to laser confocal imaging. The results were shown in Figure S2. Due to the fact that iRGD had both active targeting (capable of specifically binding to the highly expressed integrin on the surface of MCF-7 cells) and transmembrane effects, this property was endowed to SiO_2/AuNCs-iRGD, resulting in a greater accumulation of SiO_2/AuNCs-iRGD nanoparticles in MCF-7 cells and displaying strong red fluorescence. However, SiO_2/AuNCs did not possess this effect (the BSA on the surface of AuNCs cannot specifically bind to the integrin), merely showing the passive penetration effect of nanoparticles into tumor cells. Therefore, only a small amount of SiO_2/AuNCs can enter MCF-7 cells, which confirmed the binding of iRGD to SiO_2/AuNCs.

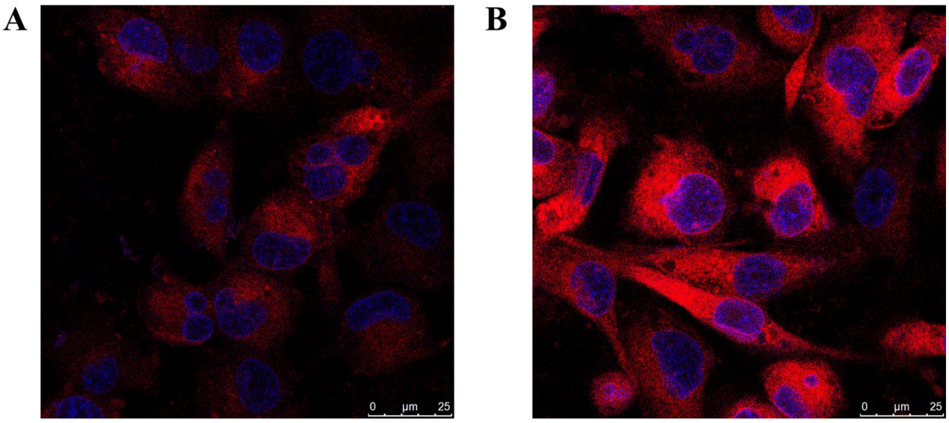

Figure S2. Laser confocal images of MCF-7 cells after incubation with SiO_2/AuNCs (A) and SiO_2/AuNCs-iRGD (B) for 2 h at 37 ℃.

3. Equivalent circuit fitting parameters obtained from EIS

Table S1. Equivalent circuit fitting parameters obtained from EIS for different samples

Sample	Rs/Ω	R_{ct}/Ω	Q/S·s^n	n	W/Ω·$s^{-1/2}$
1.Bare Au electrode	19.96	89.4	3.46×10^{-6}	0.89	1.20×10^{-3}
2.AuNPs/Au electrode	20.15	16.1	1.89×10^{-5}	0.84	1.19×10^{-3}
3.DNA-modified AuNPs/Au electrode before incubation with MCF-7 cells and DOX	23.62	386.6	2.77×10^{-6}	0.95	1.08×10^{-3}
4.DNA-modified AuNPs/Au electrode after incubation with MCF-7 cells and DOX	23.70	170.2	3.11×10^{-6}	0.96	8.63×10^{-4}
5.DNA-modified AuNPs/Au electrode after incubation with MCF-7 cells and DOX, followed incubation with cell lysis buffer	22.16	296.2	2.81×10^{-6}	0.95	1.08×10^{-3}
6.DNA-modified AuNPs/Au electrode before incubation with MCF-7 cells, SiO_2/AuNCs-iRGD and DOX	23.46	365.1	2.91×10^{-6}	0.95	1.09×10^{-3}
7.DNA-modified AuNPs/Au electrode after incubation with MCF-7 cells, SiO_2/AuNCs-iRGD and DOX	24.31	138.2	3.30×10^{-6}	0.95	1.31×10^{-3}
8.DNA-modified AuNPs/Au electrode after incubation with MCF-7 cells, SiO_2/AuNCs-iRGD and DOX, followed incubation with cell lysis buffer	22.78	298.5	2.87×10^{-6}	0.96	1.07×10^{-3}
